# Therapists’ perspectives on using brain-computer interface-triggered functional electrical stimulation therapy for individuals living with upper extremity paralysis: a qualitative case series study

**DOI:** 10.1186/s12984-022-01107-2

**Published:** 2022-11-23

**Authors:** Hope Jervis-Rademeyer, Kenneth Ong, Alexander Djuric, Sarah Munce, Kristin E. Musselman, Cesar Marquez-Chin

**Affiliations:** 1 Rehabilitation Sciences Institute, University of Toronto, Toronto, Canada; 2 The KITE Research Institute, Toronto Rehabilitation Institute, University Health Network, Toronto, Canada; 3 Department of Physical Therapy, University of Toronto, Toronto, Canada; 4 Department of Occupational Science and Occupational Therapy, University of Toronto, Toronto, Canada; 5 Institute of Biomedical Engineering, University of Toronto, Toronto, Canada

**Keywords:** Brain-computer interface, Functional electrical stimulation, Spinal cord injury, Stroke, Paralysis, Rehabilitation

## Abstract

**Background:**

Brain computer interface-triggered functional electrical stimulation therapy (BCI-FEST) has shown promise as a therapy to improve upper extremity function for individuals who have had a stroke or spinal cord injury. The next step is to determine whether BCI-FEST could be used clinically as part of broader therapy practice. To do this, we need to understand therapists’ opinions on using the BCI-FEST and what limitations potentially exist. Therefore, we conducted a qualitative exploratory study to understand the perspectives of therapists on their experiences delivering BCI-FEST and the feasibility of large-scale clinical implementation.

**Methods:**

Semi-structured interviews were conducted with physical therapists (PTs) and occupational therapists (OTs) who have delivered BCI-FEST. Interview questions were developed using the COM-B (Capability, Opportunity, Motivation—Behaviour) model of behaviour change. COM-B components were used to inform deductive content analysis while other subthemes were detected using an inductive approach.

**Results:**

We interviewed PTs (*n* = 3) and OTs (*n* = 3), with 360 combined hours of experience delivering BCI-FEST. Components and subcomponents of the COM-B determined deductively included: (1) Capability (physical, psychological), (2) Opportunity (physical, social), and (3) Motivation (automatic, reflective). Under each deductive subcomponent, one to two inductive subthemes were identified (*n* = 8). Capability and Motivation were perceived as strengths, and therefore supported therapists’ decisions to use BCI-FEST. Under Opportunity, for both subcomponents (physical, social), therapists recognized the need for more support to clinically implement BCI-FEST.

**Conclusions:**

We identified facilitating and limiting factors to BCI-FEST delivery in a clinical setting according to clinicians. These factors implied that education, training, a support network or mentors, and restructuring the physical environment (e.g., scheduling) should be targeted as interventions. The results of this study may help to inform future development of new technologies and interventions.

**Supplementary Information:**

The online version contains supplementary material available at 10.1186/s12984-022-01107-2.

## Background

For individuals living with spinal cord injury (SCI), rehabilitation strategies have been moving away from a compensatory approach towards emphasizing a restorative approach [1, 2]. Restorative approaches to rehabilitation target neuroplastic change (i.e., changes in the structure and/or function of neurons) [3] through employing task-specific practice and other principles of neuroplasticity to recover physical function [3, 4]. Interventions for other neurological conditions, including stroke, follow similar procedures to leverage neuroplasticity. Neurotechnologies (NTs) are technological devices that connect directly to the nervous system. NT can stimulate neuroplasticity through increasing dosage (e.g., number of repetitions, time exercising) and complementing hands-on or other approaches to provide therapy [5].

One recent intervention for rehabilitation of voluntary movement combines a brain-computer interface (BCI) with functional electrical stimulation (FES), two NTs. BCI-triggered FES therapy (BCI-FEST) is a combination of NT and task-specific training that has positively affected upper extremity (UE) function in individuals living with cervical SCI, or who have had a stroke [6–12]. These improvements have been supported by increased scores on clinical outcome measures, increases in range of motion (i.e., wrist), and changes in neurophysiological function indicative of neuroplastic changes (e.g., increased corticospinal excitability) in individuals with subacute SCI and chronic stroke [6, 13–15].

BCI-FES, combined with UE movement practice, has undergone feasibility testing for stroke [8, 12] and has shown promising results for UE rehabilitation, even when compared to FES alone for individuals with SCI in a randomized controlled trial [13]. Usability of the BCI-FES system for UE rehabilitation has been rated high by recipients and caregivers when studied within the home setting [16]. In this setting, recipients and caregivers also broadly discussed barriers (i.e., technical complexity of setup, lack of clinical evidence) and facilitators (i.e., seeing their hand moving, doing something useful for loved ones) to BCI-FES adoption at home. Previous research also showed that BCI-FES combined with occupational therapy is feasible for use in clinical real-world settings according to significant improvements in outcome measures [i.e., Fugl-Meyer Assessment (FMA) and Motor Activity Log 14—Amount of Use (MAL-AOU)] and therapists’ satisfaction ratings according to the Quebec User Evaluation with Assistive Technology 2.0 [17]. Participants achieved significantly improved scores on the FMA and MAL-AOU outcome measures. The level of satisfaction with the system for most therapists was above “somewhat satisfied”. BCI-FEST, which combines NT with a neurorestorative approach to therapy, has not been integrated clinically on a larger scale within healthcare systems [8, 12].

### Evaluated system and intervention

#### BCI-FEST system

Since 2016, we have developed and tested the efficacy of BCI-FEST as a tool to support task-specific training for rehabilitation of voluntary UE function in individuals with paralysis resulting from stroke and SCI. The technology is flexible enough to accommodate the requirements associated with each of these populations (e.g., practice focused on one or two upper extremities for rehabilitation after stroke or SCI, respectively), and support a therapist's clinical reasoning. In this intervention, individuals with UE paralysis practice specific skilled functional tasks that are meaningful to them, which is believed to facilitate neuroplastic change [18]. The movements are assisted by electrical stimulation and guided by a therapist. The stimulation is delivered using non-invasive electrodes and a multi-channel programmable stimulator. This device makes it possible to specify the characteristics of the stimulation (e.g., intensity) and activation sequence of each stimulation channel. The combination of careful placement of stimulation electrodes over targeted muscles and programmable features of the stimulator facilitate functional movements that are used to practice meaningful tasks during therapy. New tasks are practiced throughout the intervention, selected according to the patient’s goals, and adjusted for difficulty according to observed improvement.

The BCI is implemented as a brain switch, created to detect attempted movements following a cue from the treating therapist. The BCI is activated when the power within a user-specific EEG frequency band is sustained below an activation threshold. This threshold, as well as the time during which the power should be reduced, can be adjusted at any moment. Activation of the BCI also controls the execution of the stimulation program. Details of the system, which was specifically developed to support delivery of FES therapy, are available elsewhere [19].

#### Intervention

A typical intervention involves 40 one-hour sessions delivered three to five times each week. Each session starts by setting up the BCI and FES systems. After discussing with the person receiving therapy the tasks to be practiced, the treating therapist places the stimulation electrodes and determines the stimulation intensities for each stimulation channel. The BCI system is setup by a BCI operator—an individual with a technical background familiar with the design and operation of the system. The BCI setup includes placing the required EEG electrodes and configuring the BCI to the specific recipient (i.e., filtering the EEG to extract the band identified previously as suitable for implementing the BCI), and setting the detection threshold.

During treatment, the therapist cues the person receiving therapy to attempt the practiced task; the BCI detects the movement attempt and triggers the stimulation, the therapist then assists and guides the movement to ensure its quality. The practiced tasks can often be complex and involve multiple joints (e.g., drinking from a water bottle or using eating utensils). This process is repeated several times and the task can be changed at the discretion of the therapist or the request of the patient. In addition, the BCI operator is present throughout the entire session to make any necessary adjustments, provide support to the therapist, and answer questions about the technology.

To implement knowledge in health sciences research the Knowledge to Action (KTA) Framework is used to enhance patients’ health status. This framework is based on the definition of knowledge exchange described by the Canadian Health Services Research Foundation [20]. This definition suggests that “knowledge exchange is collaborative problem-solving between researchers and decision makers that happens through linkage and exchange. Effective knowledge exchange involves interaction between decision makers and researchers and results in mutual learning through the process of planning, producing, disseminating, and applying existing or new research in decision-making [21].” The KTA Framework incorporates two concepts: knowledge creation and the action cycle [20]. As knowledge is created, the individuals who produce it can adapt it to the needs of potential users. The action cycle leads to application of the knowledge creation.

Consulting the KTA Framework, the next step towards implementation of BCI-FEST within the action cycle is to adapt this therapy to the local context (i.e., rehabilitation hospital setting) [20]. Since BCI-FEST appears feasible for UE rehabilitation in this stage, there is an opportunity to identify barriers and facilitators that may impact its large-scale use in clinical practice in the long run. However, further efficacy testing is also necessary. Identifying barriers to BCI-FEST use should target a goal of selecting, tailoring, and implementing modifications to the therapeutic intervention. Understanding the experience of therapists delivering BCI-FEST is necessary to determine if and what change(s) need to be made to ensure long-term sustainable use in clinical practice. By examining the perspectives of physical therapists (PTs) and occupational therapists (OTs) who have delivered BCI-FEST for individuals with cervical SCI and stroke, we can determine how best to clinically implement BCI-FEST for UE rehabilitation using these therapists as delivery agents.

Our study described therapists’ perspectives on the clinical feasibility of task-specific training using BCI-FEST as an intervention for UE rehabilitation for individuals with stroke or SCI. Furthermore, we determined the perceived barriers and facilitators to providing UE therapy using BCI-FEST. Lastly, we identified if and what change(s) need to occur to promote sustainable long-term clinical use of BCI-FEST for UE rehabilitation.

## Methods

### Study design

This was a qualitative exploratory study approved by the Research Ethics Board of the University Health Network (UHN), Toronto, Canada (Additional file 2).

### Participants

We recruited PTs and OTs using purposive sampling through the KITE Research Institute, Toronto Rehabilitation Institute-UHN via e-mail. All participants were licensed to work in Canada, with several years of clinical experience in stroke and SCI rehabilitation, and had delivered BCI-FEST for the UE to at least one individual with stroke or cervical SCI as part of our earlier feasibility studies in an academic rehabilitation hospital environment [8, 12, 22]. These therapists also worked clinically with SCI and stroke populations. Each individual who received BCI-FEST participated with a therapist in three 1-h therapy sessions per week for a total of 40 sessions. All participants provided signed informed consent prior to participating in this study.

### Data collection

Researchers with a physical therapy background conducted individual interviews with each participant at their workplace following a semi-structured interview guide. We based this guide on the COM-B (Capability, Opportunity, Motivation—Behaviour) Self-Evaluation Questionnaire [23] (see Additional file 1). Each interview was conducted by one of the researchers (HJ, KO, AD) over the phone, audio-recorded, and transcribed verbatim. Transcripts were returned to the participants for comments. Some study participants had collaborated previously with one of the researchers (HJ); in which case another researcher conducted the interview. HJ maintained a reflexive journal throughout the data collection process.

**Fig. 1:**
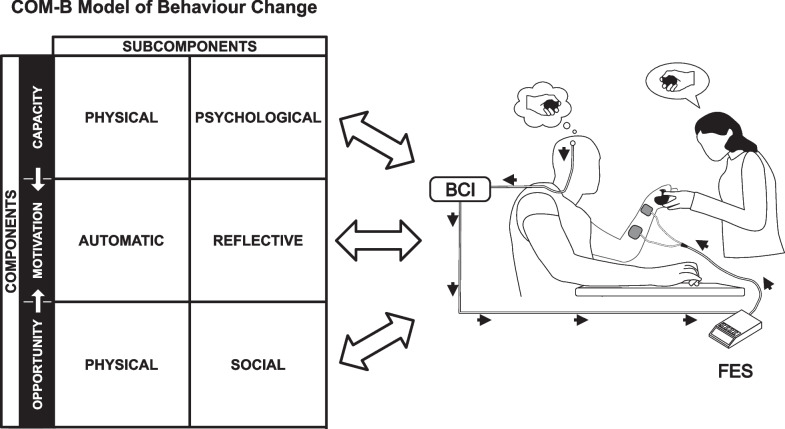
The COM-B model of behaviour change as it relates to the target behaviour: clinical use of BCI-FEST for individuals with upper extremity paralysis

The COM-B model is useful to understand how to facilitate a specific behaviour change by analysing the model’s component constructs [23]. In our case, the behaviour that we targeted was therapists’ use of BCI-FEST in clinical practice (see Fig. 1).

The first component of the COM-B, *Capability*, is divided into physical and psychological capability. Second, physical, and social subcomponents are nested under *Opportunity*. Finally, *Motivation* is comprised of reflective and automatic processes. Details of these subcomponents are provided in Table 1. Both Capability and Opportunity influence Motivation, while all three components reciprocally influence the target behaviour.

**Table 1:** Explanations of the COM-B subcomponents [23]

Subcomponent	Explanation
Physical capability	Physical capability entails the physical strength, stamina, or skill to perform BCI-FEST clinically
Psychological capability	Psychological capability entails the mental strength or stamina and knowledge, or psychological skills to engage in the necessary mental processes to perform BCI-FEST clinically
Physical opportunity	The physical opportunity to perform BCI-FEST clinically is related to the time, resources, locations, cues, and physical affordance provided by the environment
Social opportunity	Social opportunity is supplied by social cues, interpersonal influences, and cultural norms that influence our thoughts concerning a behaviour
Automatic motivation	Automatic motivation includes emotional reactions, impulses, inhibitions, drive states, reflex responses, wants and needs
Reflective motivation	Reflective motivation encompasses planning and evaluation (i.e., beliefs) about executing BCI-FEST in a clinical setting

Our possible participant pool of therapists who led BCI-FEST UE rehabilitation consisted of eight individuals. The concept of information power suggests that the more information the sample holds, the lower the number of participants required [24]. Information power is based on an assessment of five categories including: study aim, sample specificity, use of established theory, analysis strategy, and quality of dialogue. Our study aim was narrow because our research question was highly focused on the perspectives of PTs and OTs delivering therapy using a specific device. These participants were selected using purposive sampling for characteristics that were highly specific to the study aim. The COM-B and Behaviour Change Wheel (BCW) are established theories that informed both the study design and the analysis. The COM-B is part of the BCW, and they are used together to determine barriers to behaviour change and how to address them [23]. Moreover, we used a case-series analysis to allow us to detect patterns relevant to the study aim and gain in-depth information. Lastly, a researcher with extensive qualitative interview experience (HJ) conducted most participant interviews and mentored other interviewers (KO, AD). This team approach to interviewing elicited a depth of information from the interview dialogue. This analysis suggests that our sample held a large amount of information power according to all five categories; therefore, the sample size necessary to achieve the study objectives could be relatively small.

### Analysis

**Fig. 2:**
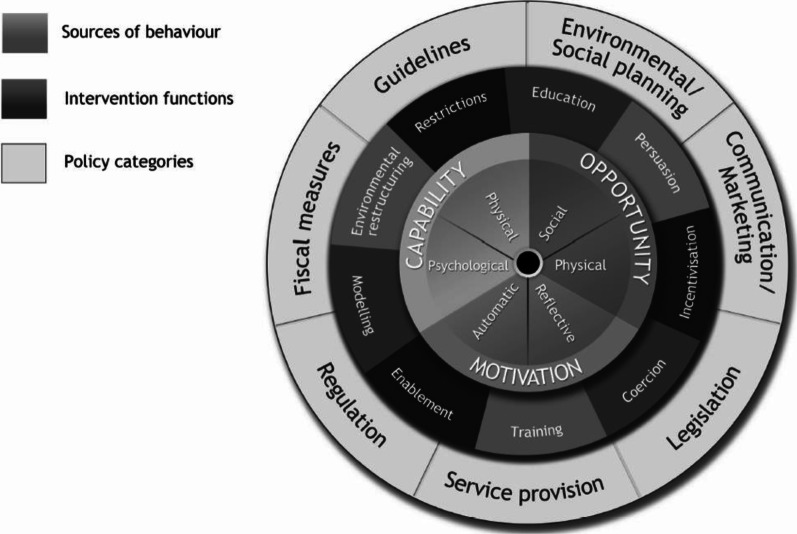
“The Behaviour Change Wheel” by Michie et al. [23] is licensed under CC BY 4.0 (https://creativecommons.org/licenses/by/4.0/)

We used a deductive content analysis technique to analyze the transcribed interviews [25]. Content analysis is used to represent the facts, provide knowledge and insight, and to give a practical guide to action [26]. This method aims to obtain a condensed, yet broad description of therapists’ perceptions about the clinical use of BCI-FEST. Deductive content analysis, also known as a directed approach, uses previous research to determine codes a priori [25, 27]. After developing a structured analysis matrix based on the COM-B model, two researchers (KO, HJ), who identified as male and female respectively, immersed themselves in the data. They coded data independently according to the matrix categories and discussed discrepancies with a third reviewer (AD), also male. Using an inductive (i.e., conventional) content analysis, themes and categories are determined directly from the text [25, 27]. In our study, we encountered some data that did not fit the deductive coding scheme; therefore, these data were used to create subthemes according to an inductive content analysis (Fig. 2).

## Results

### Participants

We interviewed three PTs and three OTs who had 360 h of combined experience delivering BCI-FEST to individuals with UE paralysis. Interviews ranged from 22 min 41 s to 30 min 43 s in duration. One therapist initially gave consent and did not respond to further contact. Another therapist was an author on this study and therefore unable to participate. All therapists identified as women.

### Content analysis

Deductive components of the analysis were based on the COM-B model of behaviour change. As described earlier, these components included (1) Capability (physical, psychological), (2) Opportunity (physical, social), and (3) Motivation (automatic, reflective). Within these components and subcomponents, inductive analysis allowed us to identify a total of eight subthemes (Table 2). These subthemes and sample quotes that exemplify each subtheme are found in the text below and in Table 2. Participant quotes are linked to the text using Quote 1 (Q1), Quote 2 (Q2), etc.

**Table 2:** Subthemes mapped to the components and sub-components of the COM-B model

	COM-B subcomponents	Inductive subthemes	Quote number	Quote
Capability	Physical	Transfer of physical ability and modality use	1	“I didn’t feel like I needed more skills in using the FES unit because it was pretty straight (forward), the FES unit.” (P6)
			2	“[Would you need to increase your physical strength or endurance?] "Not really I go to the gym.” (P5)
			3	“I wouldn’t say I have to increase my strength or even facilitation. I always get the patient into a more biomechanically advantageous position for me to do these treatments.” (P1)
	Psychological	Applying varying depths of evidence-based research to BCI-FEST	4	“I did not receive any formal training. But, of course, informal training in the sense of how the BCI works, what potentially are the benefits of using BCI-FES or only using FES.” (P3)
			5	“I brag about delivering an evidence-based intervention. So, I really am patient centred. But [patients] ask me: “Is this going to improve? How is my movement? Is this voluntary or a reflex? Is it spasticity or hypertonia?”…Your comments, your information, that you are disseminating to your patient, their family, or your other team members in rounds must be very accurate. Will you rely on science rather than: “Oh I think? Oh, maybe?” [Science] is more accurate. Consistent. So, then we can use an outcome measure.” (P5)
		Reliance on the supplementary knowledge of the BCI operator	6	“I would feel more comfortable to know that I have resources and other people to talk to if I have trouble using it or if I have a difficult case. Also, with the [BCI operator] understanding the device better in a technical way, that would be great too.” (P4)
			7	“And some patients, they were frustrated because, I think, lots of therapists and engineering…I prefer the therapist to know everything and have one less person in the scene. Less interruption because we want to satisfy the patient and get the most of it.” (P5)
Opportunity	Physical	Set-up time relative to therapy time required for BCI-FEST	8	"To be honest, I think lots of patients would benefit from (BCI-FEST) so just the barriers of setup time, having an extra person there, and whether and how effective the therapy is. (This is) what will take me one way or the other in terms of whether I choose to use the unit or not.” (P1)
			9	“I think when you are doing the therapy, you always wonder whether it was enough time; but I think when you look back on it you realize it’s tiring for the patient. If they’re really focusing, doing a good job, and putting all their mental attention to it, you probably don’t want to do it too long. Probably half an hour is a good amount of time for them to fully concentrate and focus.” (P2)
			10	“Maybe it was the study, the push, you know, rush rush RUSH. We have one hour. The patient, might have a catheter, have to, empty their bladder, then they come back. It was a rush. So, when we have someone, in this one-hour window, it didn’t allow me to treat the way I treat my patients (P5)
		Extra resources are needed to increase the efficiency and clinical feasibility of BCI-FEST	11	“In a clinical sense, outside of a study, it would be resource intensive because it requires the computer and the head setup and the FES unit, and then two people, both the engineer and the therapist, to be available at the same time, right?” (P6)
	Social	BCI-FEST therapist community development	12	If there were a group of therapists that all utilize “BCI FEST. If you could converse with them about it…It’s a new device, it’s research and I’m pretty sure at the beginning, not everyone will be using it. It (could be used) to share experiences, the good and the bad.” “I think the idea of having a group is very good and frequent communication with the group for example. So, just reach out to them: Are you using the BCI-FES?” “Do you have any questions? Do you need a call?” Just a reminder because things like that go on a shelf very easily and they stay there.” (P4)
Motivation	Automatic	Passion for technology promotes the intuitive use of BCI-FEST	13	"Oh yeah. I think that any new technology, I think that you have to get into it and practice so that it will become second nature.” (P3)
	Reflective	Knowledge from scholarly practice and inquiry innately transfers to BCI-FEST	14	“In general, I look at the patient and see where they’re at and what their goals are. Then I try to think of different technology, different treatment styles that would help them reach their goals. If BCI-FES is one of them, it’s something that I would bring up to them and see what their thoughts are.” (P1)
			15	“So doing (UE training) 1 h, 5 times per week, we are not training the rest of what (the patient) needs to work on. Maybe in the chronic stages when they want to work mostly on the hands and upper extremity function, that will be good. But in acute and subacute rehab, this is absolutely not something that I will do because it won’t do the rest (of the body) and the rest is very important.” (P4)
			16	“I think this system is going to help me to evaluate my interventions, whether they are working or not working.” (P5)

### Capability

#### Physical capability

All therapists felt that they had the physical capability to conduct BCI-FEST in a clinical setting based on their experience in a research setting. While the BCI operator applied the EEG electrodes and set up the BCI, the therapist placed the stimulation electrodes and set up the FES portion of the system. Each therapist felt that they possessed the skills needed for this task and that no additional skills were necessary (Quote 1- Q1). The subtheme we detected under the physical capability component was:

##### Transfer of physical ability and electrotherapeutic modality experience

Therapists’ prior experiences included not only their physical abilities but also their use of electrotherapeutic modalities such as FES. These modalities use different forms of energy to stimulate physiological effects and are commonly used in physical therapy practice [28, 29]. Therapists felt that these motor skills transferred directly to their physical ability to use the BCI-FES.

*“I don’t think (I needed to increase physical strength and stamina) because the type of therapy we are doing already is quite hands on and it can be physical.” (Participant 2—P2)*

Most therapists had built up their physical capabilities (i.e., strength, endurance) through working clinically prior to the research study. Others also mentioned engaging in physical activity outside of work (Q2). One therapist noted that she was used to performing similar movements already as part of her therapy practice and she employed positions that emphasized proper biomechanics when delivering BCI-FEST (Q3).

#### Psychological capability

All therapists, regardless of experience were comfortable with their mental processes (e.g., clinical reasoning) while delivering BCI-FEST, yet some were more comfortable than others with their depth of knowledge about the system and the supporting research evidence. The following subthemes were identified as part of psychological capability:

##### Applying varying depths of evidence-based research to BCI-FEST

All therapists wanted to apply evidence-based research to BCI-FEST; however, there was a range among them. Some therapists wanted basic knowledge, while others wanted deep knowledge about the system and the therapy.

*“In the beginning, like part of the learning curve, I had to dedicate a little bit more mental space into delivering BCI-FEST.” (P1).*

Therapists who had less experience with technology (i.e., FES) prior to engaging in the research study found there was a steeper learning curve compared to their peers. Even experienced therapists thought it was important to know the basics about how BCI-FES works and the potential benefits of BCI-FEST versus FEST, although this information was delivered informally (Q4). The therapists in our study defined sufficient knowledge as basic technical information about the device (including the BCI portion and BCI-FES working together). Clinically, the therapists wanted deeper and specific evidence-based knowledge (i.e., research comparing outcomes of BCI-FEST vs. FES only). They also demonstrated a need to acquire deep evidence-based knowledge to provide answers to patient’s potential questions regarding BCI-FEST(Q5).

##### Reliance on the supplementary knowledge of the BCI operator

All therapists relied on a BCI operator to set up the BCI, troubleshoot that portion of the BCI-FES system, and answer any BCI-related questions during the preliminary clinical studies.

*“There was (a BCI operator) there that was taking care of the technical aspects of (BCI-FEST), so I felt like I didn’t really need to know too much about that. Just a basic idea of how (the BCI-FES) works.” (P2).*

There was a feeling that therapists did not require in-depth knowledge about the technical aspects of the system because the BCI operator was there for the set up and session duration. Clinically, therapists felt that having a BCI operator present who understands the technical aspects of the device would help (Q6). Also, having another member of the team there for therapy to assist with difficult cases would be beneficial. Interestingly, one therapist felt that having an extra person in the room (i.e., BCI operator) frustrated the person receiving the therapy (Q7). This therapist preferred to have more knowledge about the BCI so that she could incorporate BCI-FEST by herself clinically to focus on the patient.

### Opportunity

#### Physical opportunity

Both PTs and OTs observed that more physical opportunity was needed to clinically implement BCI-FEST. They felt that a significant amount of support was needed across diverse physical factors to improve the efficiency of clinical sessions in the future. The subthemes we identified under physical opportunity were:

##### Set-up time relative to therapy time required for BCI-FEST

To set up the system, approximately 20% (i.e., 12 min) of the total therapy session time was used. As therapists became more familiar with the system, set up time decreased. Some therapists perceived that the long set-up time was a barrier to using BCI-FEST (Q8).

*“At least for the patient population that we were treating I don’t think they would be able to tolerate more than what we were delivering at the time.” (P3)*

Individuals receiving the therapy who had UE paralysis were able to tolerate active BCI-FEST for about a half hour to forty-five minutes before fatiguing as assessed by the treating therapist. This observation that set-up time was a barrier was contradicted when, on reflection, it was perceived that there was more than enough time to facilitate a therapy session despite the set-up time length (Q9). However, the research targeted BCI-FEST only; if other techniques were applied, or there were interruptions, similar to a session in a clinical environment, one hour might not be enough time (Q10).

##### Extra resources are needed to increase the efficiency and clinical feasibility of BCI-FEST

This subtheme refers to the physical resources related to BCI-FEST delivery such as personnel, facility, and technical components of the device that would facilitate clinical translation of BCI-FEST.

*“The system (has) to be more user-friendly, no flaws…It’s just a system, it has technical challenges.” (P5)*

To transition BCI-FEST to the clinic, the system would need to be updated so that it is easy to use. Therapists perceived BCI-FEST in general as resource intensive, requiring two individuals (e.g., therapist and BCI operator) to set up the individual devices (i.e., BCI on the head and FES on the UE muscles) (Q11). For clinical implementation of BCI-FEST, therapists want a device that is more user-friendly and a streamlined approach to setup and therapy delivery.

#### Social opportunity

According to our results, therapists determined that an increase in social opportunity would be necessary to facilitate clinical implementation of BCI-FEST. Therapists provided numerous examples of social support that could enable them to improve therapy delivery. The subtheme we detected as part of social opportunity was:

##### BCI-FEST therapist community development

Therapists agreed that they would need the support of others, including a community to promote the clinical use of BCI-FEST.

*“I think what helps the most is having a mentor or somebody that you can go to on the spot if you are having issues—having like an expert on staff, maybe another therapist that would be the BCI expert.” (P2).*

Therapists suggested that having a mentor within their department to help with troubleshooting the BCI-FES system would be beneficial. Many therapists felt that a support network of BCI-FEST therapists where they could share experiences would help with clinical implementation (Q12). In addition to sharing experiences, therapists could reach out to provide support and engage in frequent communication to encourage BCI-FEST use.

### Motivation

#### Automatic motivation

Therapists were comfortable with their automatic motivation for using BCI-FEST in a research setting and expanding that motivation to a larger scale clinical setting. All therapists possessed this type of motivation as evidenced by their participation in the initial BCI-FEST feasibility studies. The subtheme that we identified under automatic motivation was:

##### Passion for technology promotes the intuitive use of BCI-FEST

Therapists involved in this research were interested in using technology and most were also highly experienced in using technology clinically and/or for research studies.

*“I love technology. My PhD was on technology. I think that is the road we have to go. We clinicians have to accept, embrace and utilize (technology), but still, I feel the resistance among us.” (P5).*

Therapists who performed BCI-FEST as part of this research study were probably more passionate about technology-enriched therapy than most clinical therapists. Clinically, it will be important to practice BCI-FEST, so it becomes habitual for therapists learning this technology (Q13).

#### Reflective motivation

All therapists routinely engaged in reflective practice throughout their participation in prior BCI-FEST research and this practice would transfer to the clinical use of BCI-FEST. Reflection is the art of critically and systematically analyzing and evaluating patient abilities and goals in relation to the task demands and performance contexts. This leads to new ways of understanding and thinking in a clinical setting [30, 31]. The subtheme that we recognized under reflective motivation was:

##### Knowledge from scholarly practice and inquiry innately transfers to BCI-FEST

Reflective processes are part of therapists’ education, both at university and through continuing education. They also engage in reflection as part of their routine rehabilitation practice in all clinical settings.

*“I actually did extra work looking at different treatment techniques for stroke and spinal cord just so I had that background information.” (P2)*

Using reflective processes, therapists improved their knowledge and use of the BCI-FEST. One therapist did background research on different treatment techniques based on population. Clinically, one therapist would evaluate a patient’s goals and then suggest BCI-FEST, if appropriate (Q14), while another therapist reflected that BCI-FEST could be used as an assessment tool (Q15). Reflecting on research sessions, participants felt that BCI-FEST would not translate clinically as is (i.e., UE only focus), because therapists need to work on other patient goals as well (Q16).

## Discussion

Therapists interviewed as part of our study selected and suggested tailoring parts of BCI-FEST to move towards large scale clinical implementation. From their perspectives, BCI-FEST was both valuable and useful, but not yet fit for the broader clinical setting. Our findings revealed perceived barriers and facilitators within the COM-B model to delivering BCI-FEST clinically. Therapists felt competent in the following areas that would facilitate this therapy: physical capability, automatic motivation, and reflective motivation. However, to deliver sustainable long-term BCI-FEST clinically, all therapists felt that they would need increased social opportunity. Some therapists thought that physical opportunity should be supported further (e.g., dedicated BCI-FEST sessions), and that psychological capability should be supported with therapists new to BCI-FEST.

### The Behaviour Change Wheel (BCW) and intervention functions

The BCW is a framework based on numerous theories of behaviour change [23, 34]. The COM-B model, situated at the centre of the wheel, identifies sources of behaviour that are the most useful targets for intervention. Located in the middle portion of the wheel, intervention functions are the way that a particular intervention is characterized. Different intervention functions target different components of the COM-B. The COM-B identifies what components need to change to achieve the target behaviour while the BCW identifies what intervention functions are the most likely to stimulate that change. For example, incentivization (e.g., funding grant) could result in faster clinical implementation of BCI-FEST, if appropriate. We can apply the BCW to identify which intervention functions target the COM-B components relevant to our study to promote the clinical use of BCI-FEST.

### Social opportunity

The BCW analysis for intervention functions to improve the social opportunity for therapists to use BCI-FEST clinically align with our participants’ perspectives. Participants felt that a mentor within the department would facilitate BCI-FEST delivery, which could foreseeably help with implementation. This aligns with addressing interpersonal influences using modelling to shape therapists’ way of thinking [23, 32]. Research has shown that PTs prefer to receive intervention resources from another PT, but researchers and individuals with lived experience were also found to be acceptable [33]. Other research has also found that pairing clinicians with similar specialties and allowing mentees to give input on their coach selection and target skills have facilitated a coaching partnership [34]. Interestingly, in this same study, local champions paired the coaches and clinicians.

Therapists in our study commonly suggested a support network to facilitate clinical use of BCI-FEST. According to BCW intervention functions, creating a network would restructure the social environment and provide modelling, thus enabling therapists. In this context, modelling refers to providing an example for an individual to imitate or aspire to be like [23]. A Community of Practice (CoP) is defined as a “group of people who share a concern, a set of problems, or a passion about a topic, and who deepen their knowledge and expertise in this area by interacting on an ongoing basis” (p.4) [35]. A CoP would provide resources by facilitating learning, reflection, interaction with peers, and inducing changes in practice [36]; however, this could be a challenge when few therapists are experienced with BCI-FEST delivery.

### Psychological capability

The results from our study show that education was the intervention function that could target improving knowledge about clinical implementation of BCI-FEST. Other domains such as mental skill, mental strength and mental stamina did not require intervention. Some therapists felt the need to acquire deep evidence-based knowledge to provide answers to patients’ potential questions. Searching for evidence has been found to facilitate the implementation of evidence-based practice and having a librarian to assist with the search was helpful [37]. Acquiring “strong evidence” that was “easy-to-use” described the most valuable research for implementation.

Specifically, therapists in our study desired to understand the basics about how the system works and a comparison of BCI-FEST versus FES-only therapy. Current evidence suggests that BCI combined with electrical stimulation produces better results than FES [13, 38]. Unfortunately, there is little understanding of the neurophysiological mechanisms behind these devices. The device setup [12, 19] and feasibility [6, 13, 22, 39–42] have been documented in research to inform educational content. Ultimately, as research aggregates, educational content of training courses, or CoP resources, for example, should be updated.

### Physical opportunity

An analysis of the physical opportunity to provide BCI-FEST clinically reveals that training and restructuring the environment to provide cues and prompts can address time barriers and other physical barriers [23, 32]. Despite an average system setup time of 11 min and 13 s for studies that our therapists participated in [19], they desired to decrease this time further. However, with practice research has shown that donning times significantly decrease [16].

Interestingly, despite the perception that therapists needed more setup time for the BCI-FES device, they observed that recipients had enough therapy time considering fatigue. Fatigue is common post stroke; for example, Ingles et al. found that 88% of survey respondents (3–13 months poststroke) reported increased fatigue compared to 36% of the control group consisting of independent community-dwelling older adults who had no history of stroke [43]. Two years post-stroke, Van der Werf found the 50% of survey respondents who were stroke outpatients (≥ two years post-stroke) noted fatigue as their main complaint compared to 16% of the age-matched control group [44]. Individuals with SCI also typically experience more physical fatigue than individuals without a known pathology [45, 46]. In fact, levels of generalized fatigue are high in individuals with SCI [47, 48]. These levels were higher among outpatients with SCI if they were admitted for medical reasons, had spasticity, pain, incomplete injuries and/or were taking medication with a side effect of fatigue [47]. Many of these contributing factors fluctuate, hence they would cause energy levels to fluctuate too. Thus, depending on a patient’s current energy and fatigue levels, the individual receiving BCI-FEST may or may not be able to complete a longer therapy session. Also, studies investigating FES and muscle fatigue in individuals with SCI and those who have had a stroke have shown ways to mitigate these effects such as optimizing electrode positioning, fine-tuning parameters and stimulation patterns, and adjusting the mode and frequency of exercise training [49, 50]. Although these adjustments may reduce muscular fatigue potentially reducing overall fatigue levels, they contribute to an increase in setup time.

Restructuring the scheduling and content sessions could facilitate clinical implementation of BCI-FEST. In a typical clinical session, other therapeutic goals would be targeted (e.g., trunk control, lower extremity function); therefore, one hour may not provide enough time to include BCI-FEST. However, research has found that the highest percentage of therapy sessions for individuals with SCI are structured around one intervention [51]. These interventions often focus on hand and arm function, which is not mutually exclusive from other categories (e.g., joint mobility, manual positions, and movements). Therefore, using BCI-FEST to rehabilitate UE function could improve other goals. Additionally, restructuring the schedule could provide cues to facilitate workflow. For example, BCI-FEST scheduling could incorporate dedicated BCI sessions timed so that patients can join other forms of therapy immediately afterwards [52]. Considering these factors together, potentially no intervention function may be required, and physical opportunity may be afforded with BCI-FEST. In addition, it is possible that new technological developments will also address other therapeutic goals.

Our findings concerning BCI-FEST are applicable to implementation in inpatient or outpatient hospital rehabilitation. In acute care hospital settings, BCI-FEST may not be supported at this time. For example, previous research by Jervis Rademeyer et al. [53] found that therapists have competing interests such as patient safety and tolerance, and they desire a portable or hands-on approach to technology to support SCI rehabilitation. This approach may not apply to private practice in the community, as our study focuses on the publicly funded system.

### Limitations

There are two major limitations to this study that should be considered in future research. First, the interviews were conducted over the phone; therefore, latent content (non-verbal cues) could not be observed. An analysis of latent content was not necessary considering our research question, as the topic was not sensitive and non-verbal cues were likely congruent with therapists’ responses. Using the phone interview method as an advantage, we were able to collect data during the COVID-19 pandemic. Second, the therapists were from the same representative environment, so their perspectives may not be generalizable to clinical practice in other settings. Nonetheless, these are the only therapists we know of that have delivered task-oriented BCI-FEST incorporating multiple complex movements to individuals with UE paralysis and they represented both PT and OT perspectives.

### Implications

Future research should investigate the perspectives of individuals who receive BCI-FEST to comprehensively assess the barriers to knowledge use in the KTA process [20]. Similarly, future studies should consider the perspectives of additional therapists and other individuals (e.g., physical or occupational therapy assistants and technologists) who may be part of delivering BCI-FEST to compare with this analysis of therapists’ perspectives. It is necessary to always be aware of current barriers and facilitators to ensure a sustainable intervention; therefore, the phases of the knowledge creation and action cycles of the KTA often occur simultaneously. Consistent with KTA, our plan is to incorporate barriers and facilitators that we have currently identified to modify the intervention. This is part of the next stage of the KTA process (i.e., select, tailor, implement interventions) and is likely to be an important component towards a broader BCI-FEST implementation.

## Conclusions

Therapists’ who delivered BCI-FEST as part of upper extremity rehabilitation felt that despite facilitators currently in place, certain barriers within the COM-B prevent broad clinical implementation at this time. After completing a behavioural diagnosis of the relevant COM-B components we found that psychological capability, social opportunity, and physical opportunity need to change so that BCI-FEST can be used clinically with appropriate patients who have UE paralysis. These results may help to inform changes to BCI-FEST including the device and way that therapy is delivered.

## Supplementary Information

**Additional file 1. **Semi-structured interview guide based on the COM-B model of behaviour change self-evaluation questionnaire described in ‘The Behaviour Change Wheel: a guide to designing interventions’ by Michie, Atkins and West (2014).

**Additional file 2. **COREQ (COnsolidated criteria for REporting Qualitative research) Checklist. Checklist that describes the inclusion and location of items considered part of research for studies involving interviews or focus groups.

## Data Availability

The datasets presented in this article are not readily available because of ethical and privacy restrictions. Requests to access the datasets should be directed to the University Health Network Research Ethics Board (reb@uhnresearch.ca).
