# Supplementary material for: Therapists’ perspectives on using brain-computer interface-triggered functional electrical stimulation therapy for individuals living with upper extremity paralysis: a qualitative case series study
Source: J Neuroeng Rehabil. 2022 Nov 23;19:127. doi: 10.1186/s12984-022-01107-2 (PMC9684970; doi:10.1186/s12984-022-01107-2)
Supplement: Supplementary file 1 — Additional file 1. Semi-structured interview guide based on the COM-B model of behaviour change self-evaluation questionnaire described in ‘The Behaviour Change Wheel: a guide to designing interventions’ by Michie, Atkins and West (2014). [file 12984_2022_1107_MOESM1_ESM.pdf]

Semi-structured interview guide based on the COM-B model of behaviour change self-evaluation questionnaire described in ‘The Behaviour Change Wheel: a guide to designing interventions’ by Michie, Atkins and West (2014)

	Based on your experience delivering BCI-FEST, when it comes to you personally performing BCI-FEST for the upper extremity with a patient with stroke or SCI/D what do you think it would take for you to perform this regularly in clinical practice?<br>So, to deliver BCI-FEST to every appropriate patient would you have to....
**COM-B components**	**Interview questions and probes**
Capability	1. Know more about why it is important? (e.g. have a better understanding of the benefits of behavioural planning; have a better understanding of the technology)
	2. Feel more comfortable performing BCI-FEST? [Probe: Describe the training you received.]
	3. Increase the physical skills that BCI-FEST delivery requires?
	4. Increase mental skills, like learning how to reason effectively or ‘thinking on your feet’ to deliver BCI-FEST?
	5. Increase physical strength is required to deliver BCI-FEST?
	6. Develop mental strength, such as resilience against switching tasks too early (e.g. giving the patients enough time to initiate an upper limb movement before applying the stimulation)?
	7. Overcome physical limitations that you might have? (e.g. get around problems relating to disability)
	8. Overcome mental obstacles? (e.g. reduce unwanted feelings or temptations)
	9. Improve your physical stamina to deliver BCI-FEST?
	10. Improve your mental stamina to deliver BCI-FEST? (e.g. develop greater capacity to maintain mental effort)
Opportunity	11. Increase the amount of time need for set up? [Probe: How much time did you have to set up and complete a therapy session? Did you feel that this was enough time? Considering the set-up time, was there usually enough time to perform therapy?]
	12. Increase funding allocation for this device to be available clinically? [e.g. would the cost of this device be manageable clinically?] [Probe: Do you think that another option would work like renting?]
	13. Have more support if you wanted to implement BCI-FEST clinically at your workplace? [Probe: funding, management, personnel, supporting equipment]
	14. Improve the accessibility of BCI-FEST? [Probe: How could you make it more accessible clinically?]
	15. Be part of a group that performs BCI-FEST whether in your department or remotely across sites?
	16. Have more triggers to prompt you? (e.g. more reminders at strategic times)
Motivation	17. Feel a sense of satisfaction about delivering BCI-FEST?
	18. Feel that you need to do it enough? (e.g. care more about the negative consequences of not doing it)
	19. Believe that it would be a good thing to do (e.g. have a stronger sense that you should do it
	20. Develop better plans for doing it? (e.g. have a clearer and better developed plan for achieving it)
	21. Develop a habit of doing it? (e.g. get into a pattern of doing it without having to think)
	22. Is there anything else that you would like to mention related to your capability, opportunity or motivation that would help you to deliver BCI-FEST clinically?
